# Toxicity of dose-escalated radiotherapy up to 84 Gy for prostate cancer

**DOI:** 10.1007/s00066-023-02060-2

**Published:** 2023-03-17

**Authors:** Johannes Rosenbrock, Christian Baues, Marius Kreis, Redouane Fouassi, Eren Celik, Pia Paffenholz, David Pfister, Axel Heidenreich, Simone Marnitz

**Affiliations:** 1 Department of Radiation Oncology, CyberKnife and Radiation Therapy, Faculty of Medicine and University Hospital of Cologne, University of Cologne, Kerpener St 62, 50937 Cologne, Germany; 2 Department of Urology, Faculty of Medicine and University Hospital of Cologne, University of Cologne, Kerpener St 62, 50937 Cologne, Germany

**Keywords:** Genitourinary toxicity, Gastrointestinal toxicity, Simultaneous integrated boost, Whole gland radiotherapy, Volumetric modulated arc therapy

## Abstract

**Purpose:**

The outcome of radiotherapy (RT) for prostate cancer (PCA) depends on the delivered dose. While the evidence for dose-escalated RT up to 80 gray (Gy) is well established, there have been only few studies examining dose escalation above 80 Gy. We initiated the present study to assess the safety of dose escalation up to 84 Gy.

**Methods:**

In our retrospective analysis, we included patients who received dose-escalated RT for PCA at our institution between 2016 and 2021. We evaluated acute genitourinary (GU) and gastrointestinal (GI) toxicity as well as late GU and GI toxicity.

**Results:**

A total of 86 patients could be evaluated, of whom 24 patients had received 80 Gy and 62 patients 84 Gy (35 without pelvic and 27 with pelvic radiotherapy). Regarding acute toxicities, no > grade 2 adverse events occurred. Acute GU/GI toxicity of grade 2 occurred in 12.5%/12.5% of patients treated with 80 Gy, in 25.7%/14.3% of patients treated with 84 Gy to the prostate only, and in 51.9%/12.9% of patients treated with 84 Gy and the pelvis included. Late GU/GI toxicity of grade ≥ 2 occurred in 4.2%/8.3% of patients treated with 80 Gy, in 7.1%/3.6% of patients treated with 84 Gy prostate only, and in 18.2%/0% of patients treated with 84 Gy pelvis included (log-rank test *p* = 0.358).

**Conclusion:**

We demonstrated that dose-escalated RT for PCA up to 84 Gy is feasible and safe without a significant increase in acute toxicity. Further follow-up is needed to assess late toxicity and survival.

## Background

The outcome of radiotherapy (RT) for prostate cancer (PCA) depends on the delivered dose [1–8]. Modern techniques of external beam radiation therapy (EBRT) such as intensity-modulated radiation therapy (IMRT) [9] and image-guided radiation therapy (IGRT) [10–13] reduce the risk of side effects, allowing dose escalation compared to conventional 3D conformal radiation therapy (3D-RT). For patients with PCA, dose-escalated irradiation up to 78–80 gray (Gy) is well confirmed [6, 14–16] and studies with brachytherapy boost suggest that further dose escalation leads to better local control [17–20]. Spratt et al. demonstrated the efficacy and safety of EBRT with 86.2 Gy in a large retrospective study [21] and the FLAME study even showed an advantage of an intra-prostatic EBRT boost of up to 95 Gy over conventional IMRT with 77 Gy [22]. However, these highly sophisticated EBRT concepts have not yet gained acceptance in clinical practice. Accordingly, European Association of Urology (EAU)/European Society for Radiotherapy and Oncology (ESTRO) guidelines recommend 74 to 80 Gy for low-risk PCA and 76–78 Gy for intermediate- and high-risk PCA [23]. Barelkowski et al. developed whole-gland tomotherapy up to 84 Gy using a combination of sequential and simultaneous integrated boosts (SIB) and reported excellent oncologic outcome and toxicity data in a retrospective study of 88 patients [24]. After we applied Barelkowski’s target volume and dose concept to volumetric modulated arc therapy (VMAT) in 2016, this concept became the new standard therapy for high-risk prostate cancer patients in our institution.

The aim of the present study is to evaluate the safety of dose-escalated RT up to 84 Gy in everyday clinical practice.

## Patients and methods

In our retrospective analysis, we included all patients with PCA who were treated in our clinic with dose-escalated RT during the years 2016–2021. Patients had been previously presented in an interdisciplinary oncological conference and, depending on the risk group, informed about the possible treatment options: active surveillance, radical prostatectomy, and radiotherapy. Treatment was initiated after detailed informed consent. Depending on the risk profile, neoadjuvant and adjuvant androgen deprivation therapy (ADT) was delivered for 18 and 36 months, respectively, according to the EAU guidelines [23].

### Dose prescription and contouring

Patients underwent magnetic resonance imaging (MRI) for contouring. Patients with a risk of less than 30% for extra-prostatic extension, a risk of seminal vesicle involvement less than 10%, and a risk of lymph node involvement less than 10% according to the Memorial Sloan Kettering Center nomogram (https://www.mskcc.org/nomograms/prostate/pre-op) were usually treated with 80 Gy in two treatment steps.

**Fig. 1:**
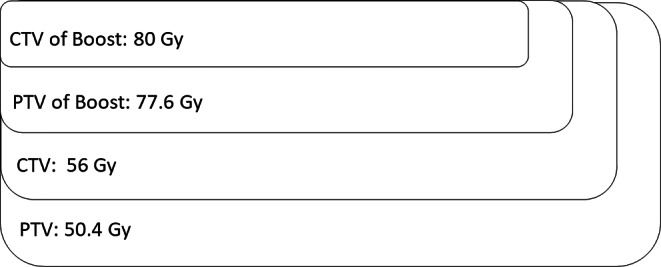
Dose prescription—80 gray (Gy); *CTV* clinical target volume, *PTV* planning target volume

In the first step, the clinical target volume (CTV) included the prostate and 5 mm of the periprostatic space. Planning target volume (PTV) encompassed this CTV with a margin of 5 to 8 mm depending on the presence of fiducials. Dorsally, the PTV was limited to 3 to 6 mm and was irradiated up to 50.4 Gy with 1.8 Gy per fraction. An SIB of 56 Gy was administered to the CTV in 2 Gy per fraction. In the second treatment step (sequential boost), the CTV only included the prostate. The PTV (for margins, see above) was treated with 21.6 Gy in 1.8 Gy per fraction and an SIB of 24 Gy in 2 Gy was administered to the CTV of the sequential boost. In total, the cumulated dose was 80 Gy (56 Gy + 24 Gy). Figure 1 gives an overview about the dose prescription in the patients treated with 80 Gy.

Patients with low-risk PCA could alternatively be irradiated with standard radiotherapy delivered up to 80 Gy in 2‑Gy single doses. In this case, the prostate was equivalent to the CTV and the PTV was created with a 5-mm (dorsal 3‑mm) margin around the CTV. Neither a simultaneous nor a sequential boost was administered.

Patients with a risk higher than 13% for extra-prostatic extension or a more than 10% risk of seminal vesicle involvement were usually treated with 84 Gy in two treatment steps, if there was an additional risk of lymph node involvement of less than 10% according to Memorial Sloan Kettering Center nomogram.

**Fig. 2:**
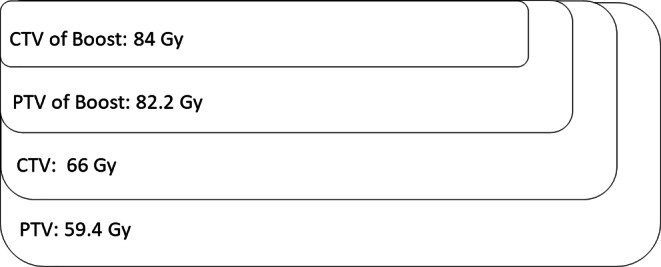
Dose prescription—84 gray (Gy) prostate only; *CTV* clinical target volume, *PTV* planning target volume

In the first step, the CTV included the prostate, the periprostatic space up to the pelvic wall, and the proximal 2 cm of the seminal vesicles. The PTV (for margins, see above) was irradiated with 59.4 Gy and a daily dose of 1.8 Gy per fraction. An SIB of 66 Gy was administered to the CTV in 2 Gy per fraction. In the second treatment step (sequential boost), the CTV only included the prostate. The PTV (for margins, see above) was irradiated with 16.2 Gy in 1.8 Gy per fraction. An SIB of 18 Gy in 2‑Gy single doses was administered to the CTV of this sequential boost. In total, the cumulated dose was 84 Gy (66 Gy + 18 Gy). Figure 2 shows the dose prescription of the patients treated with 84 Gy to the prostate only.

Current guidelines [23] are cautious in recommending prophylactic pelvic irradiation due to unclear data [25]. Nevertheless, many studies that established the combination therapy consisting of radiotherapy and ADT included pelvic irradiation [26–28]. Therefore, we discussed pelvic irradiation individually with patients who had a risk of lymph node involvement over 10% and especially greater than 15%.

**Fig. 3:**
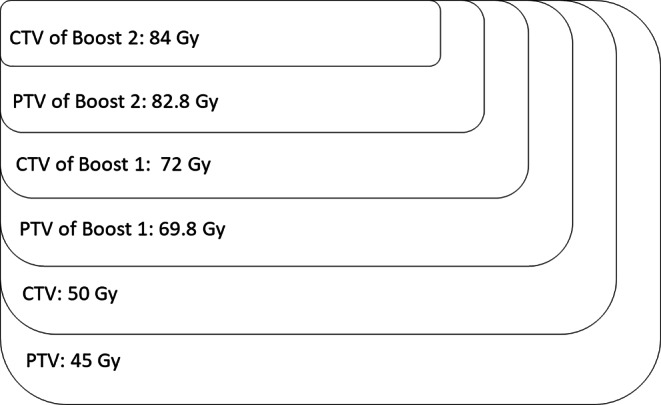
Dose prescription—84 gray (Gy) whole pelvis; *CTV* clinical target volume, *PTV* planning target volume

If pelvic irradiation was performed, it consisted of three treatment steps. In the first step, the CTV included the prostate, the periprostatic space up to the pelvic wall, the proximal 2 cm of the seminal vesicles, and the pelvic lymph nodes up to the level of the junction of L5 and S1. The PTV encompassed this CTV with a margin of 8 mm and was irradiated to 45 Gy with a dose of 1.8 Gy per fraction. To the CTV, an SIB was administered with a dose of 50 Gy in 2‑Gy single doses. In the second treatment step (first sequential boost), the CTV included the prostate, 5 mm of the periprostatic space, and the proximal 2 cm of the seminal vesicles. The PTV (5–8 mm margin, dorsally 3–6 mm) was treated with 19.8 Gy in 1.8 Gy per fraction. An SIB of 22 Gy in 2‑Gy single doses was administered to the CTV of the first sequential boost. The CTV of the second sequential boost included only the prostate. The PTV (5–8 mm margin, dorsally 3–6 mm) was radiated with 10.8 Gy per fraction. An SIB of 12 Gy in 2‑Gy single doses was administered to the CTV of the second sequential boost. Cumulatively, this resulted in 84 Gy (50 Gy + 22 Gy + 12 Gy). Figure 3 shows the 84 Gy dose prescription (including pelvis) and Table 1 summarizes the whole radiotherapy concept.

**Table 1:** Radiotherapy concept

	80 gray		84 gray without pelvis		84 gray with pelvis	
Treatment step 1	CTV1: prostate and 5 mm of the PS		CTV1: prostate, the PS up to the pelvic wall, and SV		CTV1: prostate, the PS up to the pelvic wall, SV, and the pelvic LN	
	PTV1	SIB to CTV1	PTV1	SIB to CTV1	PTV1	SIB to CTV1
Dose	50.4 Gy	56 Gy	59.4 Gy	66 Gy	45 Gy	50 Gy
Number of fractions	28	28	33	33	25	25
Treatment step 2	CTV2: prostate		CTV2: prostate		CTV2: prostate, 5 mm of the PS, and SV	
	PTV2	SIB to CTV2	PTV2	SIB to CTV2	PTV2	SIB to CTV2
Dose	21.6 Gy	24 Gy	16.2 Gy	18 Gy	19.8 Gy	22 Gy
Number of fractions	12	12	9	9	11	11
Treatment step 3	–		–		CTV3: prostate	
					PTV3	SIB to CTV3
Dose					10.8 Gy	12 Gy
Number of fractions					6	6
Dose sum	80 Gy		84 Gy		84 Gy	

*CTV* clinical target volume, *PTV* planning target volume, *PS* periprostatic space, *SV* proximal 2 cm of the seminal vesicles, *LN* lymph nodes

### Radiotherapy planning and treatment

The plans used in this study are in RapidArc (two to four arcs) and helical tomotherapy IMRT (field width 2.5 cm) technology. They were created using the Eclipse planning system (version 13.6; Varian Medical Systems Inc., Palo Alto, CA, USA) and Precision planning system (version 2.0.1.1; Accuray Precision, Sunnyvale, CA, USA).

The dose calculation for Eclipse was performed with the anisotropic analysis algorithm (AAA, version 13.6.23) and for Precision planning system, the Convolution-Superposition was used. Table 2 shows the dose constraints utilized.

**Table 2:** Dose constraints

	Dose (Gray)	Maximal volume (%)
Rectum	50	50
	60	35
	65	25
	70	20
	75	15
Dorsal half of rectal circumference	65	2
Bladder	65	50
	70	35
	75	25
	80	15
Femoral Head	50	10

The required volume coverage was 95% of the PTVs should be covered with at least 97% of the prescription dose. Irradiation was administered every workday under daily image guidance.

### Follow-up

Patients were usually monitored every 3 months by prostate-specific antigen (PSA) checks. If recurrence was suspected according to the Phoenix criteria, prostate-specific membrane antigen (PSMA) positron-emission tomography (PET) was performed to differentiate between local recurrence and distant metastases. If local recurrence was suspected, a biopsy was performed after multiparametric MRI when local treatment options were considered.

### Statistics

We evaluated progression-free survival (PFS), local recurrence-free survival (LRFS), and distant metastasis-free survival (DMFS). Moreover, we investigated acute and late genitourinary (GU) and gastrointestinal (GI) toxicity.

PFS, LRFS, and DMFS were calculated using the Kaplan-Meier method. For PFS, events included death, progression, local recurrence, and occurrence of metastases. Patients irradiated with 80 Gy or with 84 Gy were considered separately. We did not compare the treatment groups for survival data by log-rank test because the risk profiles of the groups differed substantially.

We classified adverse events that occurred within 90 days of RT initiation as acute toxicity and used the Common Terminology Criteria for Adverse Events version 5.0 (CTCAE) to evaluate acute toxicity. Acute toxicities grade ≥ 2 were assessed between the three different treatment regimens (80 Gy, 84 Gy without pelvis, 84 Gy with pelvis) using Fisher’s exact test.

Late toxicity was defined as adverse events that persisted or occurred after 90 days following initiation of RT. We used the Late Effects Normal Tissue Task Force (LENT)/Subjective, Objective, Management, Analytic (SOMA) classification [29] to assess late toxicities. Patients with a follow-up shorter than 90 days after RT start were excluded from the late toxicity assessment. Late grade ≥ 2 toxicities were evaluated using a log-rank test between the three different treatment regimens (80 Gy, 84 Gy without pelvis, 84 Gy with pelvis).

We performed the statistical analysis using IBM SPSS Statistics version 28.0.1.0 (IBM Corp., Armonk, NY, USA).

## Results

### Patients

A total of 86 patients were evaluable, of whom 24 patients had received 80 Gy and 62 patients 84 Gy (35 without pelvic and 27 with pelvic radiotherapy). The mean follow-up time for the survival data was 13.2 months (minimum 0 months, maximum 60 months). Because patents with follow-up shorter than 3 months were excluded from the analysis of long-term toxicity, the mean follow-up time here was 15.2 months (minimum 3 months, maximum 60 months). Table 3 shows the patient characteristics.

**Table 3:** Patient characteristics

		80 gray		84 gray prostate only		84 gray with whole pelvis		84 gray total	
Tumor stage	–	Number	*%*	Number	*%*	Number	*%*	Number	*%*
	1a	1	4.2	0	0.0	0	0.0	0	0.0
	1b	0	0.0	0	0.0	0	0.0	0	0.0
	1c	20	83.3	21	60.0	13	48.1	34	54.8
	2a	2	8.3	3	8.6	2	7.4	5	8.1
	2b	0	0.0	1	2.9	4	14.8	5	8.1
	2c	1	4.2	9	25.7	5	18.5	14	22.6
	3a	0	0.0	0	0.0	1	2.7	1	1.6
	3b	0	0.0	1	2.9	2	7.4	3	4.8
Gleason score	*–*	Number	*%*	Number	*%*	Number	*%*	Number	*%*
	6	15	62.5	5	14.3	0	0	5	8.1
	7a	8	33.3	17	48.6	3	11.1	20	32.3
	7b	1	4.2	3	8.6	4	14.8	7	11.3
	8	0	0.0	8	22.9	13	48.1	21	33.9
	9	0	0.0	2	5.7	7	25.9	9	14.5
iPSA	–	*Mean ng/ml*	*SD ng/ml*	*Mean ng/ml*	*SD ng/ml*	*Mean ng/ml*	*SD ng/ml*	*Mean ng/ml*	*SD ng/ml*
	–	8.1	4.3	12.2	17.5	39.1	111	24.0	74.8
D’Amico risk group	–	Number	*%*	Number	*%*	Number	*%*	Number	*%*
	Low	13	54.2	1	2.9	0	0.0	1	1.6
	Intermediate	9	37.5	19	54.3	1	3.7	20	32.3
	High	2	8.3	15	42.9	26	96.3	41	66.1
ADT use	yes	3	12.5	15	42.9	26	96.3	41	66.1
	No	21	87.5	20	57.1	1	3.7	21	33.9

*iPSA* initial prostate-specific antigen, *ADT* androgen deprivation therapy, *SD* standard deviation

### Progression-free survival

**Fig. 4:**
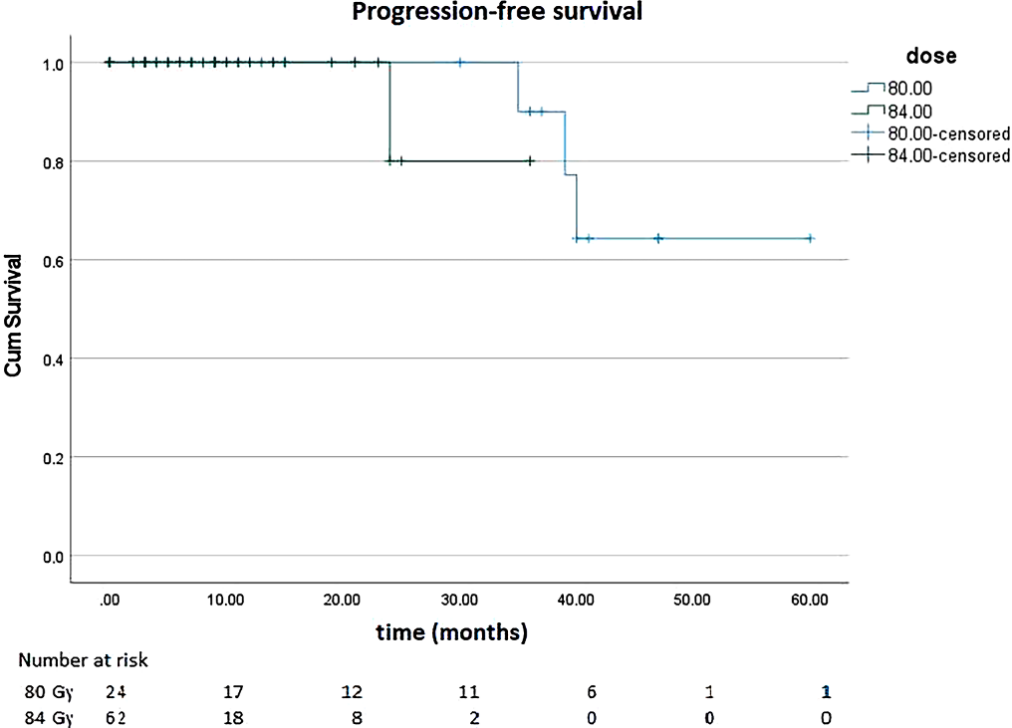
Progression-free survival of patients treated with 80 gray (Gy) and 84 Gy

With regard to PFS, 86 patients were evaluated, of whom 24 patients received 80 Gy and 62 patients received 84 Gy (35 without pelvic and 27 with pelvic radiotherapy). Among patients treated with 80 Gy, three events occurred, whereas among patients treated with 84 Gy, only one event occurred. Local recurrence occurred in one patient treated with 80 Gy. None of the patients treated with 84 Gy developed a local recurrence. Two patients, of whom one was treated with 80 Gy and one with 84 Gy, developed bone metastases during follow-up. The metastases of the patient treated with 80 Gy were not confirmed histologically and were more likely due to a renal cell carcinoma that was also detected. However, the occurrence of the metastases was nevertheless considered as an event. One patient treated with 80 Gy died because of reasons unrelated to PCA and the treatment of PCA. Figure 4 shows PFS.

### Local recurrence-free survival

**Fig. 5:**
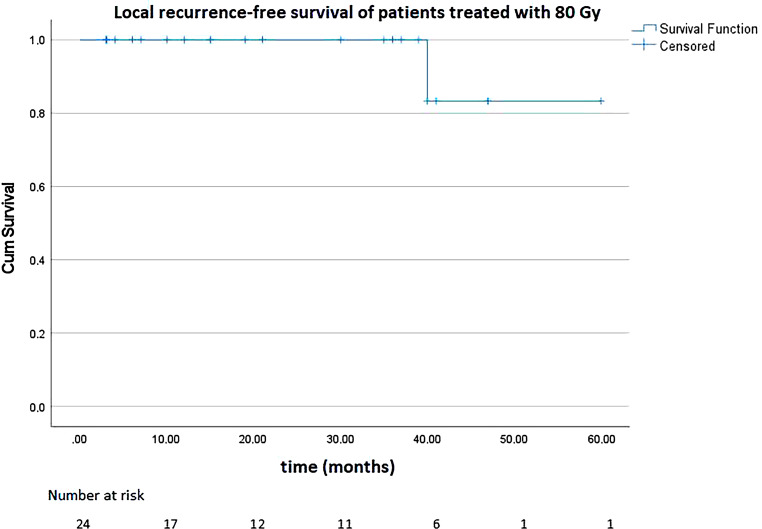
Local recurrence-free survival of patients treated with 80 gray (Gy)

With regard to LRFS, 86 patients were evaluated, of whom 24 patients received 80 Gy and 62 patients received 84 Gy (35 without pelvic and 27 with pelvic radiotherapy). Local recurrence did not occur in any of the patients treated with 84 Gy, whereas one patient with intermediate-risk PCA treated with 80 Gy developed local relapse. Remarkably, the patient who developed the local recurrence had been treated with standard radiotherapy without simultaneous boost. Figure 5 shows the Kaplan–Meier curve of LRFS of the patients treated with 80 Gy.

### Distant metastasis-free survival

**Fig. 6:**
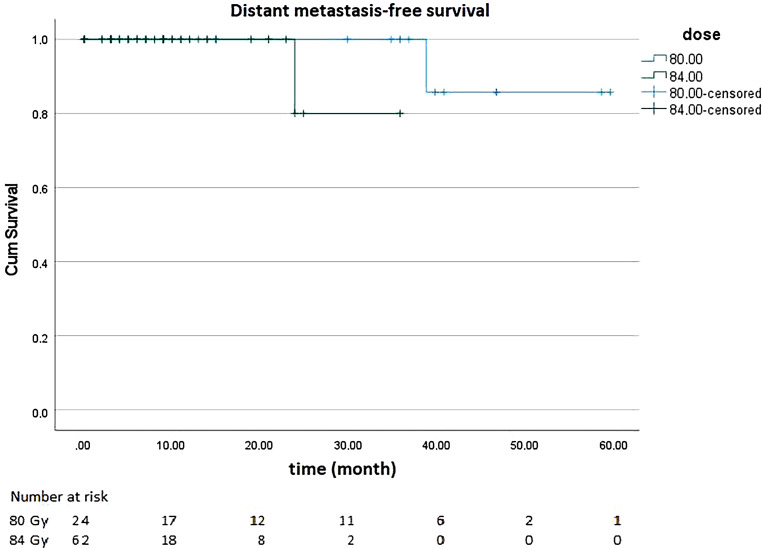
Distant metastasis-free survival of patients treated with 80 gray (Gy) and 84 Gy

With regard to DMFS, 86 patients were evaluated, of whom 24 patients received 80 Gy and 62 patients received 84 Gy (35 without pelvic and 27 with pelvic radiotherapy). One patient treated with 80 Gy developed bone metastases in the follow-up, as did one patient treated with 84 Gy including pelvic irradiation. None of the patients treated with 84 Gy excluding pelvic irradiation developed distant metastases. The metastases of the patient treated with 80 Gy were not confirmed histologically and were probably due to renal cell carcinoma, which was also found. Nevertheless, the bone metastases were considered as an event. Figure 6 show DMFS.

### Acute toxicity

Regarding acute toxicity, 86 patients were analyzed, of whom 24 patients had received 80 Gy and 62 patients had received 84 Gy (35 without pelvic and 27 with pelvic radiotherapy).

Regarding grade ≥ 2 GU toxicity, there is no significant difference in Fisher’s exact test (*p* = 0.186) when comparing patients treated to 80 Gy with patients treated to 84 Gy excluding the pelvis. In contrast, patients treated with 84 Gy including pelvic RT were significantly more likely to have grade ≥ 2 GU toxicity than patients treated with 84 Gy without pelvic RT (*p* = 0.032). Table 4 shows acute GU toxicity.

**Table 4:** Acute genitourinary toxicity

	80 gray		84 gray without pelvis		84 gray with pelvis		84 gray total	
CTCAE grade	Number	%	Number	%	Number	%	Number	%
0	10	41.70	10	28.57	8	29.6	18	29.00
1	11	45.80	16	45.71	5	18.5	21	33.90
2	3	12.50	9	25.72	14	51.9	23	37.10
3	0	0.00	0	0.00	0	0.00	0	0.00
4	0	0.00	0	0.00	0	0.00	0	0.00
5	0	0.00	0	0.00	0	0.00	0	0.00

*CTCAE* Common Terminology Criteria for Adverse Events

Regarding grade ≥ 2 GI toxicity, Fisher’s exact test showed no significant difference (*p* = 0.582) when comparing patients treated with 80 Gy to patients treated with 84 Gy excluding the pelvis. Also, there was no significant difference in grade ≥ 2 GI toxicity in patients treated with 84 Gy between those with and without pelvic irradiation (*p* = 0.510). Table 5 shows acute GI toxicity.

**Table 5:** Acute gastrointestinal toxicity

	80 gray		84 gray without pelvis		84 gray with pelvis		84 gray total	
CTCAE grade	*n*	%	*n*	%	*n*	%	*n*	%
0	17	70.80	18	51.43	18	66.7	36	58.10
1	4	16.70	12	34.29	6	22.2	18	29.00
2	3	12.50	5	14.29	3	11.1	8	12.90
3	0	0.00	0	0.00	0	0.00	0	0.00
4	0	0.00	0	0.00	0	0.00	0	0.00
5	0	0.00	0	0.00	0	0.00	0	0.00

*CTCAE* Common Terminology Criteria for Adverse Events

### Late toxicity

**Fig. 7:**
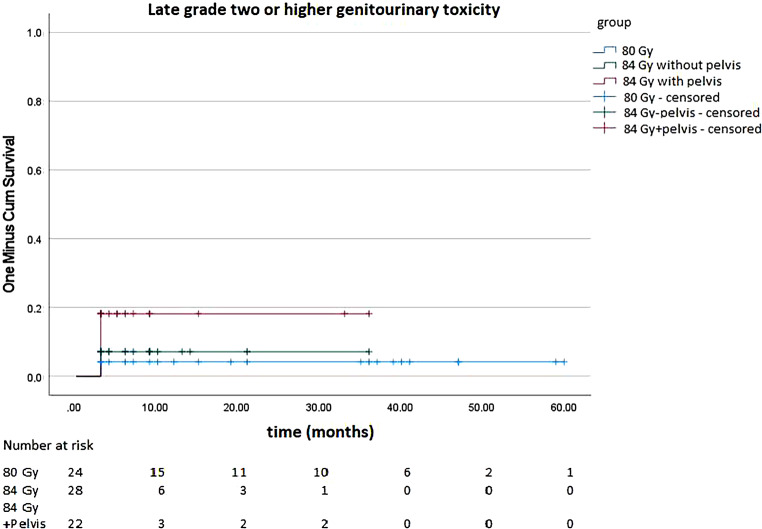
Late grade 2 or higher genitourinary toxicity of patients treated with 80 gray (Gy) and 84 Gy

Regarding late toxicity, we were able to evaluate 74 patients after exclusion of patients with missing data or without sufficient follow-up. Twenty-four of these patients were irradiated with 80 Gy and 50 patients (28 without pelvis, 22 with pelvis) were irradiated with 84 Gy. 4.17% of patients treated with 80 Gy, 7.14% of patients treated with 84 Gy excluding pelvic RT, and 18.18% of patients treated with 84 Gy including the pelvis had grade ≥ 2 GU late toxicity. There was no significant difference in log-rank-test (*p* = 0.237). Figure 7 shows GU late toxicity.

**Fig. 8:**
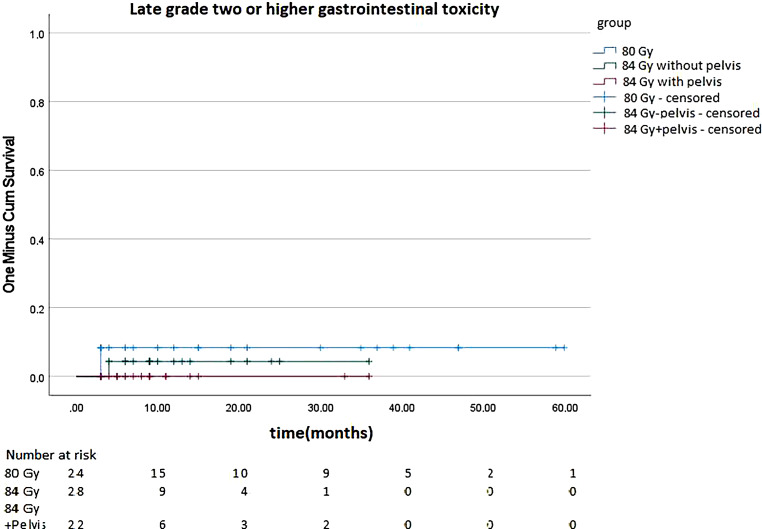
Late grade 2 or higher gastrointestinal toxicity of patients treated with 80 gray (Gy) and 84 Gy

Regarding late GI toxicity, 8.33% of patients treated with 80 Gy, 3.57% of patients treated with 84 Gy excluding pelvic RT, and 0% of patients treated with 84 Gy including the pelvis had grade ≥ 2 GI late toxicity. There was no significant difference in log-rank-test (*p* = 0.358). Figure 8 shows GI late toxicity.

## Discussion

From various studies, we have learned that the dose applied in radiotherapy of PCA has a critical impact on local control [1–8]. Modern irradiation techniques such as IMRT [9] and IGRT [11] allow dose escalation without a major increase in toxicity. Barelkowski et al. [24] and Spratt et al. [21] have published solid data for 84 and 86 Gy, respectively. However, these doses have not yet gained acceptance in clinical practice and it therefore seems necessary to generate further evidence.

Our analysis was designed to investigate the safety of dose-escalated treatment. However, due to the relatively short median follow-up time, we cannot yet make adequate conclusions on survival. Nevertheless, for the sake of completeness, we also present survival data. It is remarkable that in patients treated with 80 Gy—mainly low- and intermediate-risk patients—PFS falls below 70% after 30 months. However, due to the short follow-up period and the small number of cases, this was based on only three recurrences. Bone metastases occurred during follow-up in one patient treated with 80 Gy, but these were more likely from concurrent renal cell carcinoma. Another patient died for reasons unrelated to PCA or PCA therapy, and another patient had a local recurrence. Thus, there was only one event related to the malignancy or the necessary therapy, so this puts the poor PFS data somewhat into perspective. It is also interesting that the local recurrence occurred in a patient who was irradiated conventionally with 80 Gy, i.e., not with the SIB concept presented in Table 1. In patients irradiated with 84 Gy there was no local relapse and only one patient developed metastases during follow-up. Since 66% of patients treated with 84 Gy and as many as 96% of patients treated with whole-pelvis irradiation received a combination therapy of RT and ADT, it is not surprising that no local recurrence occurred in the short FU period. With 80% median PFS for intermediate and high-risk PCA patients, our data are comparable to those of Barelkowski (biochemical relapse-free survival 92.8% and 70.4% for intermediate- and high-risk patients, respectively) [24] and Spratt (biochemical relapse-free survival 85.6% and 67.9% for intermediate- and high-risk patients, respectively) [21]. Nevertheless, further follow-up is needed to make robust conclusions about survival.

The acute GU toxicities in our cohort can be compared with those reported in the literature (Table 6). For example, Barelkowski et al. reported 19.3%, 39.8%, 39.8%, and 1.1%, respectively, for acute GU toxicity grades 0–3 for his collective [24] and patients treated with 80 Gy or 84 Gy with and without pelvic irradiation had been included. In comparison, no acute grade ≥ 3 GU toxicity occurred in our analysis. Furthermore, grade 2 toxicities for patients treated with 80 Gy and for patients treated with 84 Gy without pelvic RT were lower than those reported by Barelkowski. Only patients with pelvic irradiation experienced more grade 2 acute toxicities than Barelkowski’s cohort (51.9% versus 39.8%). However, in his collective, pelvic RT was performed in only 12.5% of cases [24] and there was no increased acute GU toxicity compared with data from randomized trials for dose escalation up to 80 Gy [3, 6, 30]. In our study, we found a significant difference between RT with the pelvis and RT without the pelvis, but no significant difference between 80 Gy and 84 Gy. However, there was a non-significant trend that a dose increase to 84 Gy caused more acute GU toxicity. Significance would have been expected in a larger number of cases, but overall, our cohort showed a very favorable acute GU adverse event rate.

**Table 6:** Acute toxicity (grade ≥ 2)—comparison with the literature

	Genitourinary (%)	Gastrointestinal (%)
80 gray without pelvis	12.5	12.50
84 gray without pelvis	25.7	14.30
84 gray with pelvis	51.9	11.1
Barelkowski et al. (80–84 gray) [19]	40.9	12.5
Zietman et al. (79.2 gray) [3]	50.0	57.0
Beckendorf et al. (80 gray) [6]	17.5	19.5
Peeters et al. (78 gray) [25]	55.0	51.0

Regarding acute GI toxicity, the results were also similar to those of Barelkowski, who reported grade 1 and 2 GI toxicities in 29% and 11% of patients, respectively [24]. In our patients treated with 84 Gy, grade 1 and 2 GI toxicities occurred in 29% and 12.9%, respectively. Those who were treated with 80 Gy had lower GI toxicity (grade 1: 16.7%; grade 2:12.5%) and grade 3 or higher GI toxicities did not occur, neither in Barelkowski’s study nor in ours. In the randomized “80 Gy” studies by Beckendorf et al. [6] and Peeters et al. [30], acute GI toxicities were considerably higher than in our series; in particular, grade 3 toxicities also occurred (range 4–5.9%) and this is probably due to the 3D-RT primarily used in these studies. Overall, we were able to achieve lower GI toxicities using the modern RT technique despite further dose escalation to 84 Gy. This also applies to the proton boost study of Zietmann et al. in which no grade 3 GI toxicities occurred but, nevertheless, significantly more grade 2 toxicities (57%) [3].

Due to the short mean follow-up time of only 15 months, we cannot make valid statements on long-term toxicity at this time. Nevertheless, in order to not miss unexpected high long-term toxicities at an early timepoint, we recorded them. Because of the short follow-up period, our data show few late GU toxicities compared with data published in the literature. In our analysis, 4.2% of patients treated with 80 Gy and 7.1% of patients treated with 84 Gy without pelvis irradiation had grade ≥ 2 GU late toxicity and 18.8% of the patients who received pelvic radiotherapy had grade ≥ 2 GU late toxicity. In comparison, Barelkowski et al. reported a rate of 23.8% of grade ≥ 2 GU late toxicity in a pooled analysis of patients treated with 80 Gy and 84 Gy [24] and Spratt et al. reported a rate of 21.1% [21]. Also, compared to the grade ≥ 2 late GU toxicity rate of randomized dose escalation studies up to 80 Gy (range 21–26.9%) [3, 6, 30], the observed toxicity appears to be quite low. Comparable to our late GU toxicity is the 4% rate reported by Pahlajani et al. for patients treated with ≥ 80 Gy [7]. Our low rate of GU late toxicity is certainly due to the shorter follow-up compared to the other studies. From Spratt’s data, late toxicity increases steadily during the first 10 years [21], so our follow-up time (mean 15.2 months) is clearly too short for a definitive assessment of late toxicity.

Nevertheless, our data show that especially patients without pelvic irradiation have an excellent acute GU toxicity profile compared to published series. Spratt et al. demonstrated that acute toxicities have a high predictive value with respect to late toxicity [21]. Therefore, based on our low acute GU toxicity, we can assume that our toxicity rate will increase later on, but not seriously compared to the other studies. This assumption is supported by the fact that our data show no increase during the follow-up period.

With regard to late GI toxicity, we also cannot make a valid statement at this time due to the short mean follow-up time of only 15 months. However, in order to not miss unexpected high late GI toxicities at an early timepoint, we nevertheless collected the data. In our cohort, the rate of grade ≥ 2 GI toxicity was 3.6% for the patients treated with 84 Gy excluding pelvic RT and 0% for the patients treated with pelvic RT. In contrast, however, patients treated with 80 Gy had higher GI toxicity with a proportion of 8.3%. Barelkowski et al. reported a rate of 15.8% for grade ≥ 2 GI toxicity in their pooled patient population [24]. In the study of Spratt et al., 4.4% of patients had grade 2 or higher GI late toxicity [21] and in the study of Pahlajani et al., 7% of the patients treated with ≥ 80 Gy [7].

The studies that provide the rationale for dose escalation up to 80 Gy suggest a higher late GI toxicity (range for grade ≥ 2 GI toxicity: 18–41.7% [3, 6, 30]). However, Spratt et al. also demonstrated a strong association between acute GI toxicity and late GI toxicity [21], and since our acute GI toxicity is low, especially in comparison to Beckendorf et al. [6], Peeters et al. [30], and Zietmann et al. [3], it is reasonable to assume that the expected increase in late toxicity will not be drastic. This would be explained by modern irradiation techniques compared to 3D-RT.

Another method of dose escalation is the combination of EBRT and HDR brachytherapy. Hoskin et al. were able to show that this combination is significantly superior to hypofractionated non-dose-escalated EBRT in terms of recurrence-free survival, and there was no difference with regard to long-term toxicity [31]. However, the EBRT regimen with 55 Gy in 20 fractions corresponds to an EQD2 of only 65.9 Gy based on an α/β ratio of 1.8 Gy. Regarding this, the standard arm is not comparable to the dose-escalated normofractionated RT regimens discussed here (EQD2 ≥ 80 Gy) and to the now established standard of moderate hypofractionation (CHHip trial, 60 Gy in 20 fractions [32]; EQD2 of 75.9 Gy). Morris et al. demonstrated a significant advantage of a combination of EBRT (46 Gy in 23 fractions) and LDR brachytherapy (minimal peripheral dose of 115 Gy) over EBRT alone (78 Gy in 29 fractions) in terms of biochemical progression-free survival [19], but more grade 3 GU late toxicity occurred after the combination (18.4% versus 5.2% *P* < 0.001) [33].

To our knowledge, a randomized trial evaluating combination therapy including brachytherapy versus dose-escalated RT > 80 Gy does not exist. However, Spratt et al. were able to demonstrate an advantage of the combination of EBRT and brachytherapy over dose-escalated EBRT (86.4 Gy) in terms of recurrence-free survival in a retrospective series in which higher acute GU toxicity occurred with combination therapy, but no higher rate of long-term toxicities. According to the authors, many patients were irradiated using IGRT without fiducial markers, which may have influenced the accuracy of EBRT [20]. Overall, this study clearly demonstrates the potential of dose-escalated RT with brachytherapy. Nevertheless, there is a lack of randomized trials demonstrating superiority of the combination of brachytherapy with EBRT compared to dose-escalated EBRT with > 80 Gy.

Alternative to the whole-gland dose-escalated RT used in our study, dose escalation by intraprostatic boost is an option. In the FLAME study, a 5-year biochemical disease-free survival of 92% was achieved in a population of mainly high-risk patients. In terms of late GU and GI toxicity, there was only a trend toward more toxicity without significance compared to the standard 77 Gy treatment arm (GU toxicity grade ≥ 2: 28% versus 23%; GI toxicity grade ≥ 2: 13% versus 12%) [22]. Nevertheless, the concept of intraprostatic EBRT boost has yet to become established in clinical practice. The authors themselves criticize the interobserver variability in contouring the GTV, although the study shows excellent results despite this variability. However, it is unclear whether this has a decisive influence outside of a clinical study.

## Conclusion

Intra-prostatic EBRT boost will certainly be used more frequently in the future and brachytherapy shows promising results. Nevertheless, whole-gland EBRT, which has been used successfully for decades, will continue to play a significant role in clinical practice. In this context, our data show that dose escalation above 80 Gy is feasible and safe with appropriate techniques such as IMRT and daily IGRT. Further follow-up is needed to evaluate survival and late toxicity.
